# Mallard Hybridization With Domesticated Lineages Alters Spring Migration Behavior and Timing

**DOI:** 10.1002/ece3.70706

**Published:** 2024-12-30

**Authors:** Nicholas W. Bakner, Nicholas M. Masto, Philip Lavretsky, Cory J. Highway, Allison C. Keever, Abigail G. Blake‐Bradshaw, Ryan J. Askren, Heath M. Hagy, Jamie C. Feddersen, Douglas C. Osborne, Bradley S. Cohen

**Affiliations:** ^1^ College of Arts and Sciences Tennessee Technological University Cookeville Tennessee USA; ^2^ Cornell Lab of Ornithology Cornell University Ithaca New York USA; ^3^ Department of Biological Sciences University of Texas at El Paso El Paso Texas USA; ^4^ Illinois Natural History Survey, Forbes Biological Station–Bellrose Waterfowl Research Center, Prairie Research Institute University of Illinois at Urbana‐Champaign Havana Illinois USA; ^5^ Division of Agriculture Experiment Station and Arkansas Forest Resources Center University of Arkansas Monticello Arkansas USA; ^6^ U.S. Fish and Wildlife Service, Region 6 Habitat and Population Evaluation Team Bismarck North Dakota USA; ^7^ Migratory Gamebird Program Tennessee Wildlife Resources Agency Nashville Tennessee USA

**Keywords:** behavior, game‐farm, genetics, hybridization, mallard, migration, waterfowl

## Abstract

Introgressive hybridization, the interbreeding and gene flow between different species, has become increasingly common in the Anthropocene, where human‐induced ecological changes and the introduction of captively reared individuals are increasing secondary contact among closely related species, leading to gene flow between wild and domesticated lineages. As a result, domesticated‐wild hybridization may potentially affect individual fitness, leading to maladaptive effects such as shifts in behavior or life‐history decisions (e.g., migration patterns), which could influence population demographics. In North America, the release of captive‐reared game‐farm mallards (*Anas platyrhynchos*) for hunting has led to extensive hybridization with wild mallards, altering the genetic structure in the Atlantic and Mississippi flyways. We aimed to investigate differences in spring migratory behavior among 296 GPS‐tagged mallards captured during winter in Tennessee and Arkansas with varying levels of hybridization. Despite relatively low levels of genetic introgression of game‐farm genes, mallards with higher percentages of game‐farm ancestry exhibited later departure and arrival times, shorter migration distances, and a tendency to establish residency at lower latitudes. Specifically, for every 10% increase in game‐farm genetics, mallards departed 17.7% later, arrived 22.1% later, settled 3.3% farther south, and traveled 7.1% shorter distances during migration. These findings suggest that genetic introgression from game‐farm mallards influences migratory behavior, potentially reducing fitness, and contributing to population declines in wild mallards. Our study presents a need for understanding how domestic hybridization effects fitness and behavioral change of other species.

## Graphical abstract

Domesticated‐wild hybridization between game‐farm and wild mallards ( *Anas platyrhynchos* ) may impact migratory behavior and fitness. In this study, we analyzed spring migration data from 296 GPS‐tagged mallards in Tennessee and Arkansas, finding that higher levels of game‐farm ancestry were associated with delayed departure and arrival times, shorter migration distances, and settlement at lower latitudes. Our study presents a need for understanding how game‐farm mallards are influencing breeding activities and understanding regional effects of hybridization.

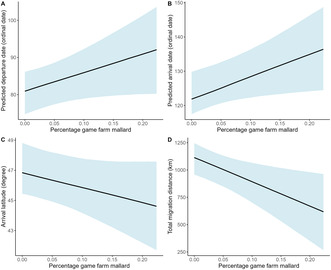

## 1 Introduction

Introgressive hybridization—the crossbreeding and transfer of genes between individuals of different species—is a widespread phenomenon (Avise 1994; Rhymer and Simberloff 1996; Mallet 2005). Hybridization can result in various outcomes: maintaining species boundaries (Lemmon et al. 2007; Roe and Sperling 2007), creating hybrid zones (Harrison 1993), or genetic swamping (process of rare taxa being replaced by hybrids) of one or both interacting taxa (Allendorf et al. 2005; Roberts et al. 2010). The likelihood of these outcomes are generally dictated by levels of genetic differentiation and hybrid viability (Todesco et al. 2016). For example, a hybrid zone is expected if hybrids are viable but less fit in either parental species' primary niche space, while genetic swamping of one parental species can occur if hybrids are equally or more fit than the parental taxa (Todesco et al. 2016; Thompson et al. 2021). Genetic swamping can also occur due to human‐induced manipulation both indirectly as a result of ecological transformation and/or directly by consistent input of domestic‐bred individuals into a wild population (Vernesi et al. 2003; Crispo et al. 2011).

Hybridization can have significant biological consequences, including effects on species' behavior. Inheritance of genetic information can cause behavioral changes in foraging strategies (De Santis et al. 2021), mating behavior (Feiner et al. 2024), migration (Helbig 1991; Bolnick, Caldera, and Matthews 2008), and resource selection (Cushman et al. 2024). In avian populations, hybridization events have been observed to influence song characteristics, leading to alterations in mate attraction, and territorial defense behaviors (Grant and Grant 1992). Similarly, hybridization in some fish species can affect migratory behaviors, leading to shifts in spawning grounds (Bolnick, Caldera, and Matthews 2008). Furthermore, genetic introgression can disrupt established patterns of social behavior, impacting group dynamics, and cooperative strategies within species (Rhymer and Simberloff 1996; Maag et al. 2024). Such alterations in behavior can have profound impacts on the fitness and survival of individuals, as well as the long‐term evolutionary trajectories of populations.

In the Anthropocene, human‐induced ecological changes and introduction of captively reared individuals are increasing the frequency of secondary contact among closely related species (Hendry, Gotanda, and Svensson 2017; Millette et al. 2020), resulting in extensive gene flow (Pelletier and Coltman 2018). While the introduction of captive‐reared individuals can enhance genetic diversity in threatened populations, especially for rare species, it also introduces risks of hybridization that may disrupt the genetic structure and adaptive potential of wild populations (Robert 2009). Among such interactions, human‐mediated introgressive hybridization between wild and domesticated lineages is increasingly common (Delibes‐Mateos et al. 2008; Champagnon et al. 2012; Blanco‐Aguiar, Ferrero, and Dávila 2022). Domesticated × wild hybrid zones are often facilitated by the escape or purposeful release of captive‐reared species for recreational purposes (Randi 2008; Barbanera et al. 2010). The consequences of anthropogenic hybridization to wild populations can be detrimental, including loss of genetic integrity and fitness outcomes, but they are often overlooked in management and policy decisions (Laikre et al. 2010). Consequently, there is a growing need to verify and monitor such interactions to provide informed conservation plans regarding potential adaptive consequences for wild populations and their evolutionary trajectories (Keller and Waller 2002; Crespel et al. 2021; Blanco‐Aguiar, Ferrero, and Dávila 2022).

The capacity to reliably establish a wild individual's ancestry allows for the identification of ecological, behavioral, and/or morphological characters that are being impacted by prevalent gene flow (Rhymer and Simberloff 1996; Allendorf et al. 2001). Waterfowl (Order Anseriformes; ducks, geese, and swans) exhibit some of the highest hybridization rates among all wild bird populations (Grant and Grant 1992; McCarthy 2006). Mallards ( *Anas platyrhynchos* ) are the most numerous and widespread waterfowl species (Kulikova et al. 2005; Baldassarre 2014). The annual release of captive‐reared mallards has been a widespread practice for over a century in North America—predominantly in the Atlantic Flyway—primarily for recreational hunting opportunities (Heusmann 1974; Osborne, Swift, and Baldassarre 2010; Lavretsky et al. 2020; Champagnon et al. 2023). The game‐farm mallard breed was assumed to be sedentary like most other domesticated breeds, and therefore were released *en masse* with the expectation that they would not interact or breed with wild mallard populations (Stanton, Soutiere, and Lancia 1992; Lavretsky et al. 2020). However, extensive genetic surveillance has proven this assumption inaccurate. Game‐farm mallards survive long enough with sufficient mobility that their presence has resulted in extensive hybridization, fundamentally altering our understanding of the genetic structuring of mallard across North America (Lavretsky et al. 2019, 2020, 2023; Lavretsky, Janzen, and McCracken 2019).

It is hypothesized that an influx of maladaptive traits from game‐farm mallard releases may contribute to observed population declines of wild mallards in the Atlantic Flyway and Great Lakes region of the Mississippi Flyway (Lavretsky et al. 2023; Schummer et al. 2023). However, it remains unclear what level of game‐farm genetic introgression into wild settings produces maladaptive traits (Tufto 2017), and which traits mechanistically reduce fitness outcomes (Lavretsky et al. 2023). For example, bill and wing morphology of game‐farm mallards are shorter with more widely spaced lamellae than wild counterparts (Champagnon et al. 2010), which may reduce foraging efficiency and long‐distance flight capacity, respectively; introgression of such traits may be maladaptive in wild settings, particularly during time periods or life stages when greater mobility is beneficial, such as during migration (Gurd 2007; Söderquist et al. 2014). Conversely, game‐farm mallards and game‐farm × wild hybrids may be uniquely adapted to anthropogenic landscapes and show affinity for urbanized spaces, where hybridization could lead to improved fitness outcomes (Lavretsky et al. 2023).

The objective of our research was to investigate mechanistic differences in spring migratory behavior among mallards with varying levels of wild ancestry in the Mississippi Flyway. Our approach involved the use of new‐generation genomic sequencing combined with GPS‐GSM telemetry to assess how genetic origin affected spring migration behaviors such as destinations, duration, distance, frequency of migratory flights, and proclivity for urban environments (Osborne, Swift, and Baldassarre 2010; Söderquist, Gunnarsson, and Elmberg 2013). We predicted that mallards with higher levels of game‐farm ancestry would depart for and arrive to the breeding grounds later, halt migration farther south, use stopovers more frequently and for longer durations, have shorter migration distances but longer overall migration durations, and show greater selectivity toward urban environments. These predictions are based on previous studies suggesting that domesticated mallards, particularly those with game‐farm ancestry, tend to exhibit traits, such as shorter wings and greater sedentism, which may influence their migration patterns, stopover behavior, and settling in urban landscapes (Stanton, Soutiere, and Lancia 1992; Champagnon et al. 2010; Lavretsky et al. 2023). Cumulatively, effects manifesting in the spring would be more likely to impact the breeding season and ultimately contribute to fitness than effects measured during fall migration and winter (Anteau and Afton 2004; Stafford et al. 2014).

## 2 Methods

### 2.1 Mallard Capture and Auxiliary Marking

**FIGURE 1:**
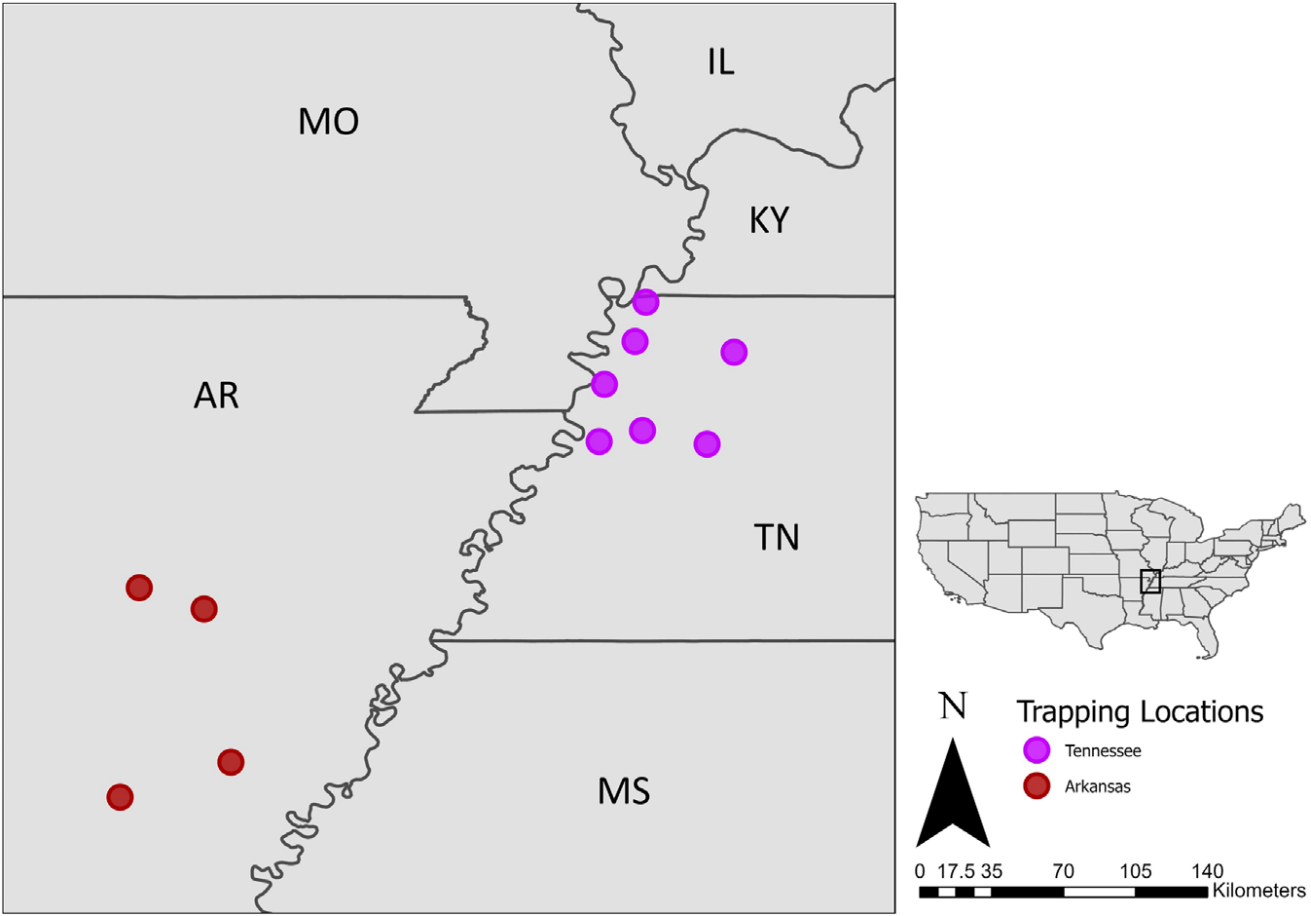
Trap site locations of mallards ( *Anas platyrhynchos* ) in Arkansas and Tennessee during 2019–2023.

We captured adult and juvenile male and female mallards using a combination of swim‐in traps, confusion traps, and rocket‐nets at four locations in Arkansas and eight locations in Tennessee from November through February 2019–2023 (Sharp and Smith 1986; Dieter, Murano, and Galster 2009; Figure 1). We banded all mallards with a United States Geological Survey (USGS) federal aluminum tarsal band. We determined age and sex of mallards based on cloacal inversion, wing plumage, and bill color (Carney 1992). Hereafter, we refer to age‐classes as juveniles or adults. We extracted ~0.01 mL of blood from the brachial artery or caudal tibial vein and stored blood at −80°C until ready for DNA extraction (Teitelbaum et al. 2023).

We attached 15‐ or 20‐g solar rechargeable and remotely programmable, OrniTrack Global Positioning System‐Global System for Mobile transmitters (GPS‐GSM; Ornitela, UAB Švitrigailos, Vilnius, Lithuania) to mallards weighing ≥ 1000 g (i.e., ≤ 2.5% of total body mass) to ensure deployment package remained below recommended body weight limits (3%–5%; Fair et al. 2010). We attached transmitters via dorsally mounted body harnesses made of automotive moisture‐wicking elastic ribbon (McDuie et al. 2019). Completed harnesses had two body loops which were knotted and sealed with cyanoacrylic glue above the keel and across the abdomen of the bird (McDuie et al. 2019). Total package of GPS‐GSM transmitter and harnesses at time of deployment weighed approximately 17–22 g. All capture and handling procedures of ducks were in accordance with Institutional Animal Care and Use Committee protocols for Tennessee Technological University #19‐20‐002, and University of Arkansas Monticello #05172021, and authorized under Federal Banding Permits #05796 and #23825, respectively.

### 2.2 Molecular Methods

DNA was extracted from whole blood, following manufacturer's protocols for the Qiagen DNA Easy Blood and Tissue Kit (Qiagen, Valencia, CA, USA). DNA integrity was based on the presence of a high‐molecular weight band as determined with gel electrophoresis using a 1% agarose gel (Graham et al. 2015).

Double digest restriction‐site associated DNA (ddRAD‐seq) fragment libraries were prepared following protocols in DaCosta and Sorenson (2014, also see Lavretsky et al. 2015) using *Sbf*I and *Eco*RI restriction enzymes with a modified bead‐based size‐selection protocol (Hernández et al. 2021). Libraries were pooled in equimolar concentrations and sent for 150 base pair (bp), single‐end sequencing on an Illumina HiSeq X (Novogenetics LTD). Previously published raw ddRAD sequences for the same samples representing mallards, game‐farm mallards, and Khaki Campbell (domestic breed) used in mtDNA analyses also served as nuclear references, and were included in following bioinformatics steps (Lavretsky, Janzen, and McCracken 2019; Lavretsky et al. 2020). All new Illumina raw reads are deposited in the National Center for Biotechnology Information's Sequence Read Archive (http://www.ncbi.nlm.nih.gov/sra; BioProject *TBD*, accession numbers *TBD*).

Bioinformatics included de‐multiplexing raw‐Illumina reads using the *ddRADparser.py* script of the BU ddRAD‐seq pipeline (DaCosta and Sorenson 2014) based on perfect barcode/index matches. Custom in‐house Python scripts (Python scripts available at https://github.com/jonmohl/PopGen; see Lavretsky et al. 2020) were then used to automate sequence filtering, alignment, and genotyping using a combination of trimmomatic (Bolger, Lohse, and Usadel 2014), burrows wheeler aligner v. 07.15 (bwa; Li and Durbin 2011), and samtools v. 1.7 (Bolger, Lohse, and Usadel 2014). All sequences were aligned to a recently published and chromosomally assembled wild North American mallard genome (Lavretsky et al. 2023). VCF files were further filtered for any base‐pair missing > 10% of samples that also included a minimum base‐pair depth of 5× (i.e., 10× per genotype) and quality per base PHRED scores of ≥ 30 using vcftools v. 0.1.15 (Danecek et al. 2011).

### 2.3 Telemetry Data Processing

We interpreted the spatial scale of migration events and stopovers by first estimating a probability‐density function of cumulative log‐transformed step‐lengths and then identifying natural breaks in the smoothed distribution of step‐lengths (Beatty et al. 2014). We interpreted step‐lengths < 0.25 km as movement within wetland complexes, step‐lengths 0.25–50 km as local‐regional movements, and step‐lengths ≥ 50 km as migration events (Dittmer et al. 2024; Highway et al. 2024; Masto et al. 2024). These distances were similar to previous research examining stopover and staging areas of dabbling ducks (Sullivan et al. 2018; Teitelbaum et al. 2023).

We considered spring migration to have begun when individuals exceeded a pre‐specified latitude that depended on their wintering origin (i.e., Tennessee or Arkansas; Beatty et al. 2013; Clements et al. 2022). Specifically, departure latitudes were defined as the northern‐most GPS location for mallards within each capture state (Arkansas and Tennessee) during January, a time when ducks were no longer migrating farther south (Schummer et al. 2010; Masto et al. 2022). We excluded migration initiations that occurred before 1 February and after 31 May. Migration initiation thresholds were 35.904° N and 36.982° N for Arkansas and Tennessee, respectively. Individuals finished spring migration when they (1) exceeded 43° N and (2) established residency by remaining within a 50 km diameter circular buffer for ≥ 10 days (Krementz, Asante, and Naylor 2011; Masto et al. 2024). We selected the earliest location within an established residency to demarcate the end of spring migration and the beginning of breeding activities (e.g., wetland prospecting).

To quantify spring migration behavior, we calculated total migration distance (km), migration duration (days), number of stopovers (*n*), and stopover duration (h) for each individual. We calculated total migration distance as the sum of step‐lengths between migration start and residency establishment. Likewise, migration duration was the time between migration start and residency establishment. We considered a stopover to be any geographical area between migration initiation and residency establishment when a bird stayed within a 50 km circular buffer for ≥ 24 h (Hupp et al. 2011). We quantified stopover duration as the total elapsed time between the first and last GPS location within the 50 km circular buffer for each stopover. We imputed zeros for stopover duration when an individual never stopped before establishing residency.

To assess how genetic ancestry affected mallard proclivity to settle near urbanized areas, we used Natural Earth version 4.0.0, which is a publicly available land cover database, from which we extracted urban areas (visible built‐up zones and generalized urban boundaries derived from recognized global datasets) for the United States and Canada using the *rnaturalearth* package in R version 4.3.3 (R Core Team 2024; Massicotte and South 2023). We then calculated the distance to urban area using the Euclidean distance tool in ArcGIS 10.8 (Environmental System Research Institute Inc., Redlands, CA, USA) keeping the raster layer at a 30 m resolution. For each individual we then extracted distance from the arrival location (i.e., the earliest GPS location associated with residency establishment) to the closest urban area.

### 2.4 Population Structure and Individual Ancestry

We used a dataset of independent bi‐allelic autosomal ddRAD‐seq single nucleotide polymorphisms (SNPs), with singletons removed across analyses of population structure. We completed all analyses without a priori information on population or species identity. We used vcftools v. 0.1.15 (Danecek et al. 2011) to extract bi‐allelic SNPs, and then plink v. 1.9 (Purcell et al. 2007) to filter for singletons (i.e., minimum allele frequency [MAF] ≥ 0.0015), any SNP missing ≥ 10% of data across samples, and linkage disequilibrium (LD). A significant linkage disequilibrium correlation factor (*r* ^2^) > 0.5 resulted in randomly excluding 1 of 2 ddRAD‐seq SNPs.

Population structure was visualized implementing a Principal Components Analysis (PCA) in PLINK v. 1.9 (Purcell et al. 2007) and calculating co‐ancestry matrix coefficients with the fineRADstructure program (Malinsky et al. 2018). The co‐ancestry matrix is based on the distribution of identical or nearest neighbor haplotypes among samples with recent co‐ancestry emphasized by rare SNPs (Kimura and Ohta 1973), and thus, an increase in these SNPs corresponds with relatedness. The fineRADstructure program was run with a burn‐in of 100,000 iterations, followed by 100,000 Markov chain Monte Carlo (MCMC) iterations, and tree building using default parameters. The co‐ancestry matrix was visualized using R scripts fineradstructureplot.r and finestructurelibrary.r (http://cichlid.gurdon.cam.ac.uk/fineRADstructure.html). Additionally, individual assignment probabilities (*Q* values) were estimated with the program ADMIXTURE v. 1.3 (Alexander, Novembre, and Lange 2009; Alexander and Lange 2011; Shringarpure et al. 2016), including standard errors based on 100 bootstrap replicates for each evaluated *K* population model. Each ADMIXTURE analysis was ran with a 10‐fold cross‐validation, and with a quasi‐Newton algorithm employed to accelerate convergence (Zhou, Alexander, and Lange 2011). Each analysis used a block relaxation algorithm for point estimation and terminated once the change (i.e., delta) in the log‐likelihood of the point estimations increased by < 0.0001. We evaluated a *K* population model of three when evaluating the entire dataset, and *K* population model of two when excluding Khaki Campbell. The latter was done to evaluate for potential individual assignment changes when including two domestic lineages versus one. Standard errors were based on 100 bootstrap replicates per ADMIXTURE analysis. In general, we expected hybrids to have multi‐population co‐ancestry and *Q* value assignments. For ADMIXTURE, *Q* scores and respective standard errors were evaluated whether they overlapped ≥ 98% population assignment that we considered to represent a genetically pure parental, whereas those individuals assigned to multiple genetic clusters determined to be as hybrids.

### 2.5 Statistical Analyses

We fitted Bayesian logistic regression models in the *brms* package in R to evaluate the relative influence of game‐farm introgression (percentage of game‐farm ancestry; percent hybridization relative to pure wildtype) on migratory performance and urban selection (Bürkner 2017). Specifically, we fit eight univariate models each with separate response variables including (1) departure and (2) arrival dates, (3) arrival date latitude, (4) number of stopovers used, (5) length of time at a stopover, (6) distance to urban areas, (7) total migration distance, and (8) migration duration. We treated all response variables as a Gaussian distribution, with the exception of number of stopovers (modeled with a Poisson distribution) and stopover duration (modeled with a negative binomial distribution). Our models included a unique identification number for each individual and migration year as a random effect to account for variation among individuals and to accommodate any correlations, thereby preventing pseudoreplication (Hurlbert 1984; Kéry and Schaub 2012). The only predictor variable in all models was the percentage of game‐farm ancestry for each individual. We standardized our predictor variable (percentage of game‐farm ancestry) by subtracting the mean and dividing by two standard deviations prior to modeling to improve model fit and interpretation (Gelman 2008). We computed four MCMC chains for 10,000 iterations, discarding the first 4000 iterations as a burn‐in (Gelman and Rubin 1992), and set the adapt_delta to 0.99 to ensure stable sampling (Bürkner 2017). All estimated parameters had $\hat{R}$ < 1.1 indicating that all chains converged (Gelman 2004). We calculated 90% credible intervals (CrI) that provided a metric of uncertainty. We interpreted support for biologically meaningful effect if CrIs surrounding percentage of game‐farm ancestry did not overlap zero. When the CrI was centered around zero (with an equal distribution on both sides of zero), the effect estimate was close to zero, and the probability of direction was less than 89%, we considered the predictor variable to have strong support for no effect. We considered CrIs that did not overlap zero with a probability of direction (pd) ≥ 89% as showing moderate support for an effect (Makowski et al. 2019; Makowski, Ben‐Shachar, and Lüdecke 2019).

## 3 Results

### 3.1 Population Structure and Individual Ancestry

DNA was extracted from whole blood drawn from 321 to 197 mallards from Tennessee to Arkansas, respectively. A total of 111,560 base‐pairs (bp) were recovered across chromosomes that met our sequencing coverage and missing data criteria for the 663 genotyped samples. An average sequencing depth of 131 sequences and range of 13–218 sequences per locus were recovered across samples.

**FIGURE 2:**
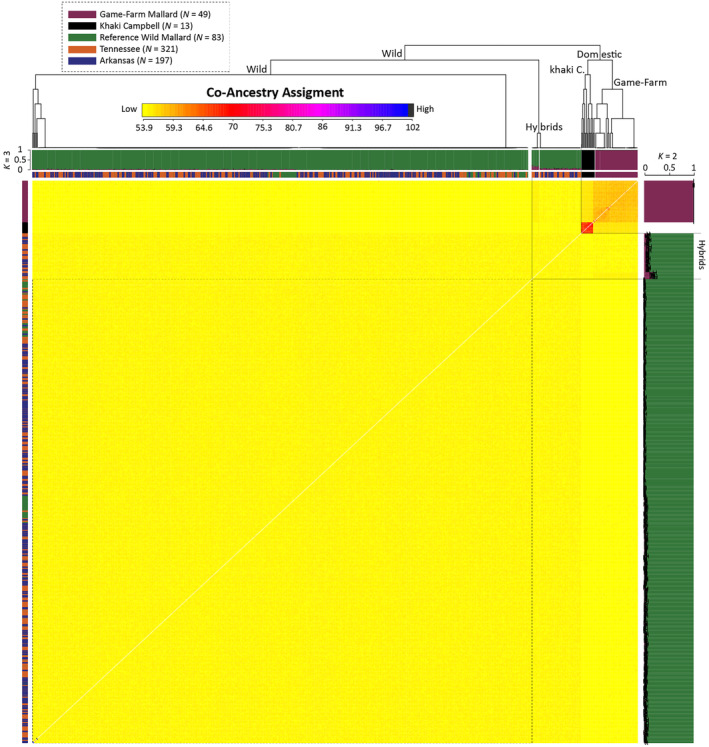
Nuclear population structure analyses based on 36,250 independent bi‐allelic nuclear SNPs assayed across 663 samples comprising our study samples, as well as reference Khaki Campbell, wild and game‐farm mallards. Population structure was assessed based on a fineRADstructure co‐ancestry matrix, and ADMIXTURE assignment probabilities obtained for all samples at a *K* of 3 or just known game‐farm and wild mallards at a *K* of 2. Note that standard errors obtained from 100 bootstrap replicates are overlaid on the ADMIXTURE analysis of known game‐farm and wild mallards at a *K* of 2.

Population structure analyses of all 663 samples were based on 36,250 (*N* = 37,235) independent bi‐allelic ddRAD‐seq SNPs. Plotting the first two components of the PCA explained 31% of the variation and provided three clear genetic groups distinguishing between wild mallards, game‐farm mallards, and Khaki Campbell (Figure 2). The same three genetic clusters were also recovered in our co‐ancestry matrix (Figure 2), and ADMIXTURE estimated assignment probabilities under a *K* of three population model (Figure 2). In addition, all reference samples falling into their respective genetic groups across analyses; wild Tennessee and Arkansas mallards were either assigned to the wild mallard genetic cluster or an admixture between wild and game‐farm mallards. To ensure that ADMIXTURE estimated individual assignment probabilities were not biased, we excluded Khaki Campbell for a dataset of 648 samples and 37,203 independent bi‐allelic ddRAD‐seq SNPs, and analyzed it under a *K* population model of two. Doing so yielded near identical *Q* values as compared to the analysis of the entire dataset under a *K* population model of three. Importantly, when aligning samples based on their location on the co‐ancestry matrix, we found two clusters of 54 wild mallards from Tennessee and Arkansas that fell outside other wild mallards in the co‐ancestry dendrogram, have slightly elevated mixed co‐ancestry between wild and game‐farm mallards, and with *Q* value standard deviations that do not overlap ≥ 98% wild mallard genetic ancestry. Conversely, we found that all wild mallards, including our reference wild mallards, fell within a single co‐ancestry matrix, with respective *Q* value standard deviations that overlapped, indicating ≥ 98% wild mallard genetic ancestry. Thus, we characterized those 54 samples as wild × game‐farm mallard hybrids, with the remaining Tennessee and Arkansas mallards as genetically wild. We consider all of the putative hybrids as late‐generational backcrosses given the lowest wild mallard ancestry assignment was 77% among the 54 samples. Regardless, our characterization resulted in a slightly higher hybrid prevalence among Tennessee (*n* = 38 ~12%) than Arkansas (*n* = 16 ~8%) samples.

### 3.2 Migration Behaviors and Chronology

We monitored 296 mallards that initiated spring migration, with 95 in Arkansas (25 females and 70 males), and 201 in Tennessee (92 females and 109 males). We obtained 2 years of spring migration data from 41 of those individuals. Hence, we modeled 337 (*n* = 110 and 227 for Arkansas and Tennessee, respectively) migratory tracks for which all individuals were genetically‐vetted.

**FIGURE 3:**
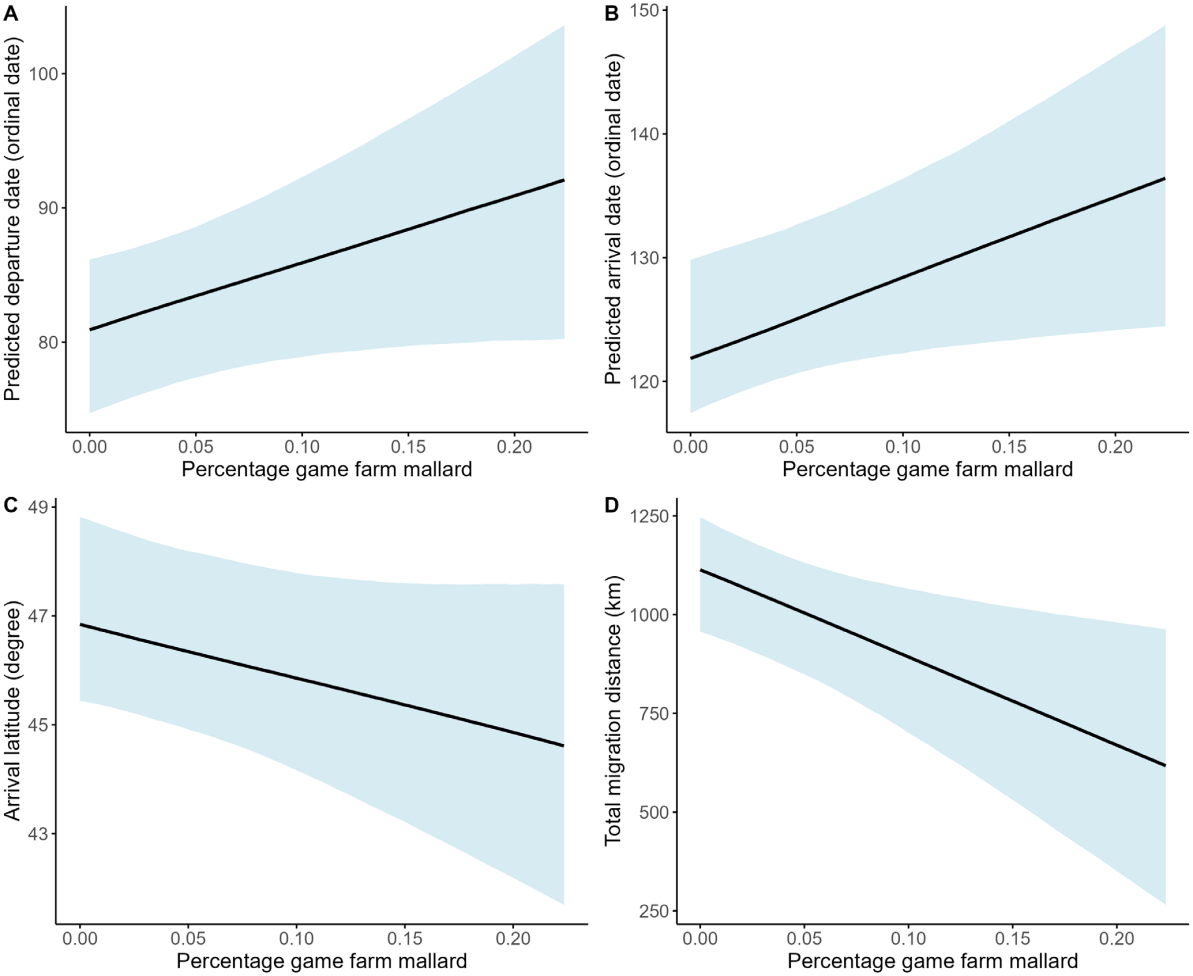
(A) Estimated spring migration departure dates, (B) arrival dates, (C) arrival latitudes (degrees), and (D) total spring migration distance (km) as a function of percentage game‐farm mallard for 337 migration tracks made by 296 mallards ( *Anas platyrhynchos* ) captured in Arkansas and Tennessee during 2019–2023. Light blue shading represents the 90% credible intervals.

Generally, mallards with a higher percentage of game‐farm ancestry departed later (*β* = 1.62, 90% CI: −0.05–3.29, pd = 94.52%; Figure 3A), established residency later (*β* = 2.02, 90% CI: 0.28–3.74, pd = 97.19%; Figure 3B), established residency at lower latitudes (*β* = −0.33, 90% CI: −0.73–0.07, pd = 91.33%; Figure 3C), and traveled less total migration distance (*β* = −70.77, 90% CI: −122.86 to −18.45, pd = 98.67%; Figure 3D). Specifically, for every 10% increase in game‐farm genetics, mallards had a 17.7% later departure date, 22.1% later arrival date, established residency in breeding locales 3.3% farther south, and a 7.1% decrease in total distance traveled during migration. We did not find statistical association between the amount of game‐farm ancestry with the number of stopovers, duration at stopovers, duration of migration, and establishment of residency closer to urban areas (Table 1).

**TABLE 1:** Parameter estimate (percentage game‐farm mallard) movement behavior model for 337 migration tracks from 296 mallards captured in Arkansas and Tennessee during 2019 and 2023.

Model	*β*	SE	CrI	pd
Arrival date	2.02	1.05	0.28–3.74	97.19
Arrival latitude	−0.33	0.24	−0.73–0.07	91.33
Departure date	1.62	1.01	−0.05–3.29	94.52
Distance to urban	90.29	8.15	−77.71–104.39	78.46
Migration duration	0.42	1.34	−1.76–2.64	62.22
Migration total	−70.77	31.62	−122.86 to −18.45	98.67
Migration duration	0.42	1.34	−1.76–2.64	62.22
Number of stopovers	−0.01	0.07	−0.12–0.10	56.84
Stopover duration	7.50	13.43	−14.48–29.75	71.01

*Note:* Model refers to the response variable in each model. Shown are regression coefficients (β), standard error (SE), 90% credible intervals (CrI) and probability of direction (pd).

## 4 Discussion

Recent genetic analyses have revealed a geographic trend in the prevalence of feral game‐farm and game‐farm × wild mallard hybrids, with prevalence decreasing from east to west, and closely aligning with areas of game‐farm mallard releases *en masse* (Lavretsky et al. 2023). Concordant with previous studies, genetic ancestry assignments recovered our samples to cluster with or between wild and game‐farm mallards, with none showing any association with our alternative domestic lineage, Khaki Campbells (Figure 2). Generally, the proportions of individuals with game‐farm ancestry that we recovered were higher than those reported for lower Mississippi Alluvial Valley (MAV; Davis et al. 2022) but lower than those from the Great Lakes Region (Schummer et al. 2023). Together, ours and the aforementioned studies suggest genetic sub‐structuring of migratory mallard populations in the Mississippi Flyway and highlight a critical need for further investigation into the factors influencing possible metapopulation dynamics.

Mallards with higher percentages of game‐farm ancestry exhibited altered migratory behaviors, including delayed departure and residency establishment, reduced migration distances, and settlement at lower latitudes (Figure 3). Changes in migration timing and locality can impact reproductive performance and eventually influence population dynamics (Aebischer et al. 1996; Gunnarsson et al. 2006; Bauer et al. 2008). For example, late arrivals to breeding grounds can result in lower reproductive success due to a shortened breeding season (Lozano, Perreault, and Lemon 1996; Bell et al. 2024), reduced forage (Sergio and Newton 2003; Lok et al. 2017), increased temperatures (Skagen and Adams 2012; Alves et al. 2013), and decreased renesting propensity (Prop, Black, and Shimmings 2003; Newton 2008). Furthermore, habitat mismatches or rearing young during periods of higher predation risk may occur when avian species arrive later to their breeding grounds (Lank et al. 2003; DeGregorio et al. 2016). Although wild mallards typically select optimal breeding times, hybrids appear to settle in areas of lower latitudes at later arrival dates. We posit that hybrids are unable to migrate to northern latitudes either due to physical limitations or because they experience reduced survival rates in those regions (Arnold and Martin 2010). Consequently, we hypothesize that interbreeding between wild and game‐farm mallards is potentially reducing North American mallards' realized niche space. As with hybrid bills being transformed to being shorter and wider (Halligan 2024) and with lowered lamellar density (Champagnon et al. 2010) that has resulted in reduced feeding efficiency (Halligan 2024), artificial selection may have also changed wing, muscle, or other physiological features that reduce their capacity to migrate (Champagnon et al. 2023). Future research would benefit from whole‐genome analyses in which association studies can be performed to determine the genetic underpinnings of these traits, shedding light into how much of the variation observed among these admixed populations is due to genetic ancestry versus environmental changes.

Stopovers are essential for refueling and rest during migration (Seewagen, Guglielmo, and Morbey 2013; Linscott and Senner 2021). The frequency and duration of stopovers were similar between wild and hybrid mallards, but wild mallards migrated farther, suggesting greater efficiency. Given the reduced foraging efficiency of game‐farm × wild mallard hybrids (Halligan 2024; Champagnon et al. 2010; Söderquist et al. 2017, Champagnon et al. 2023), hybrids may halt their migration due to an inability to accumulate the necessary body reserves to complete migration (Newton 2006; Ramenofsky and Wingfield 2006).

Although game‐farm × wild mallard hybrids are often associated with urban environments (Lavretsky et al. 2023), we found no evidence of game‐farm hybrids ending their migration closer to urban environments compared to wild mallards. Spatial barriers, specifically at breeding sites, contribute most to total reproductive isolation (Matsubayashi and Katakura 2009; Lackey and Boughman 2017). The breakdown of habitat isolation during breeding periods can lead to extensive hybridization (Taylor et al. 2006; Takimoto 2009; Elmer 2019; Ravinet et al. 2021). For instance, male farm‐raised red fox ( *Vulpes vulpes* ) dispersed away from urban areas and were the primary agents of gene flow to pure populations, whereas females remained near urban areas (Sacks, Brazeal, and Lewis 2016). This overlap may suggest a mechanism for game‐farm mallards to pair bond with wild types, facilitated by strong spatial overlap, further promoting hybridization. We acknowledge that the general lack of highly backcrossed or simply feral mallards may impact our ability to fully evaluate the cost of hybridization, as maladaptive traits of game farm mallards may have already been selected against in the lineages of late‐generation hybrids that were sampled in this study. Thus, future work will require increased sampling across the spectrum of hybrids that are known to occur in North America to understand the full effect of maladaptive traits introduced to wild mallard through game‐farm mallard introgression (Lavretsky et al. 2023).

The introgression of game‐farm ancestry into wild mallard populations presents significant conservation challenges, particularly in the context of the Anthropocene. Human activities, including the continued release of hundreds of thousands of game‐farm mallards annually since the early 1900s, have led to increased hybridization, impacting the genetic integrity and adaptive potential of wild mallard populations (Lavretsky et al. 2023). Despite lower levels of hybridization at our study sites compared to those in the Atlantic Flyway (Lavretsky 2021) or Great Lakes (Luukkonen 2024), we still detected differences in migratory behavior. In areas with greater number of hybrid or increased game farm × wild mallard hybridization proportions, hybrids are known to select urban landscapes (Osborne, Swift, and Baldassarre 2010). However, this study demonstrates strong overlap in breeding areas between wild and game‐farm hybrid mallards from wintering areas with relative low frequency of hybridization. The use of traditional breeding areas by game‐farm × wild mallard hybrids in addition to prior evidence of urban affinity, suggests greater plasticity in breeding habitats and habits of game‐farm × wild mallard hybrids. Hence, more comprehensive studies focusing on the selection of breeding areas should be conducted to understand the dispersal patterns of game‐farm mallard hybrids and mechanisms for introgression. Furthermore, our data supports the need to limit the stocking of animals from agricultural lineages to minimize impacts on wild species behavior.

## Author Contributions

**Nicholas W. Bakner:** conceptualization (equal), data curation (equal), formal analysis (lead), methodology (equal), validation (lead), visualization (lead), writing – original draft (lead), writing – review and editing (equal). **Nicholas M. Masto:** conceptualization (equal), data curation (equal), formal analysis (equal), investigation (equal), writing – review and editing (equal). **Philip Lavretsky:** conceptualization (equal), data curation (equal), investigation (equal), methodology (equal), supervision (equal), visualization (equal), writing – review and editing (equal). **Cory J. Highway:** conceptualization (equal), data curation (equal), investigation (equal), visualization (equal), writing – review and editing (equal). **Allison C. Keever:** conceptualization (equal), formal analysis (supporting), investigation (equal), writing – review and editing (equal). **Abigail G. Blake‐Bradshaw:** conceptualization (equal), data curation (equal), investigation (equal), writing – review and editing (equal). **Ryan J. Askren:** conceptualization (supporting), data curation (equal), investigation (equal), writing – review and editing (equal). **Heath M. Hagy:** conceptualization (equal), funding acquisition (equal), writing – review and editing (equal). **Jamie C. Feddersen:** conceptualization (equal), funding acquisition (equal), writing – review and editing (equal). **Douglas C. Osborne:** conceptualization (equal), funding acquisition (equal), project administration (equal), writing – review and editing (equal). **Bradley S. Cohen:** conceptualization (equal), funding acquisition (equal), investigation (equal), project administration (equal), writing – review and editing (equal).

## Conflicts of Interest

The authors declare no conflicts of interest.

## Supporting information


Appendix S1.

## Data Availability

The data that support the findings of this study are openly available in GitHub at https://github.com/nwb74172/Genetics‐Mallard‐Migration.git. Data and code additionally provided as Appendix S1.
